# MRI measurements of vessel calibre in tumour xenografts: Comparison with vascular corrosion casting

**DOI:** 10.1016/j.mvr.2012.08.001

**Published:** 2012-11

**Authors:** Jake S. Burrell, Robert S. Bradley, Simon Walker-Samuel, Yann Jamin, Lauren C.J. Baker, Jessica K.R. Boult, Philip J. Withers, Jane Halliday, John C. Waterton, Simon P. Robinson

**Affiliations:** a CR-UK & EPSRC Cancer Imaging Centre, Division of Radiotherapy and Imaging, The Institute of Cancer Research, 15 Cotswold Road Sutton, Surrey, SM2 5NG, UK; b Henry Moseley X-ray Imaging Facility, The School of Materials, The University of Manchester, Oxford Road, Manchester, M13 9PL, UK; c Centre for Advanced Biomedical Imaging, Department of Medicine and Institute of Child Health, University College London, 72 Huntley Street, London, WC1E 6DD, UK; d R&D Personalised Healthcare & Biomarkers, AstraZeneca, Alderley Park, Macclesfield, SK10 4TG, UK

## Abstract

Vessel size index (R_v_, μm) has been proposed as a quantitative magnetic resonance imaging (MRI) derived imaging biomarker in oncology, for the non-invasive assessment of tumour blood vessel architecture and vascular targeted therapies. Appropriate pre-clinical evaluation of R_v_ in animal tumour models will improve the interpretation and guide the introduction of the biomarker into clinical studies. The objective of this study was to compare R_v_ measured in vivo with vessel size measurements from high-resolution X-ray computed tomography (μCT) of vascular corrosion casts measured post mortem from the same tumours, with and without vascular targeted therapy. MRI measurements were first acquired from subcutaneous SW1222 colorectal xenografts in mice following treatment with 0 (n = 6), 30 (n = 6) or 200 mg/kg (n = 3) of the vascular disrupting agent ZD6126. The mice were then immediately infused with a low viscosity resin and, following polymerisation and maceration of surrounding tissues, the resulting tumour vascular casts were dissected and subsequently imaged using an optimised μCT imaging approach. Vessel diameters were not measurable by μCT in the 200 mg/kg group as the high dose of ZD6126 precluded delivery of the resin to the tumour vascular bed. The mean R_v_ for the three treatment groups was 24, 23 and 23.5 μm respectively; the corresponding μCT measurements from corrosion casts from the 0 and 30 mg/kg cohorts were 25 and 28 μm. The strong association between the in vivo MRI and post mortem μCT values supports the use of R_v_ as an imaging biomarker in clinical trials of investigational vascular targeted therapies.

## Highlights

► Non-invasive quantitation of vessel calibre in tumour xenografts in vivo ► Assessment of tumour vessel calibre response to a vascular disrupting agent ► Generation of vascular corrosion casts from the same tumours imaged by MRI ► Quantitation of vessel calibre from corrosion casts by microCT ► Excellent agreement between the in vivo MRI and post mortem microCT vessel calibres

## Introduction

Susceptibility contrast magnetic resonance imaging (MRI) can be used for the non-invasive determination of vessel size (R_v_, μm) and fractional blood volume (fBV, %) (Tropres et al., 2001). These quantitative imaging biomarkers have been exploited in vivo to evaluate incipient tumour vascular architecture and function (Douma et al., 2010; Farrar et al., 2010; Kostourou et al., 2003; Lemasson et al., in press; Remmele et al., 2011; Robinson et al., 2003b, 2008; Wade and Kozlowski, 2007), and tumour response to anti-vascular therapies (Beaumont et al., 2009; Howe et al., 2008; Lemasson et al., 2011; Nielsen, 2007; Ungersma et al., 2010; Valable et al., 2008; Walker-Samuel et al., 2012). For the determination of R_v_, the MRI transverse relaxation rates R_2_* and R_2_ are measured before and after the intravenous injection of an ultrasmall superparamagnetic iron oxide (USPIO) particle contrast agent, which consists of a highly paramagnetic iron oxide crystal core surrounded by a stabilising shell (Jung and Jacobs, 1995). USPIO particle contrast agents remain intravascular for the duration of the MRI acquisition, and cause R_2_* and R_2_ to become faster in tissue surrounding blood vessels that are perfused at the time of injection. The ratio of the resulting changes in the relaxation rates (ΔR_2_*/ΔR_2_) can be related to a weighted mean vessel size, R_v_, via a mathematical model of tumour blood vessel geometry (Tropres et al., 2001; Yablonskiy and Haacke, 1994). The fractional blood volume can also be estimated from the same mathematical model, using the change in R_2_* after injection of USPIO contrast agent.

Ex vivo analysis of tumour vasculature can be attained via vascular corrosion casting, which involves injecting a vascular network with a low viscosity monomer resin, which completely perfuses the vascular tree, including the micro-capillaries (Konerding et al., 1989). Shortly after injection, the resin polymerises into a solid acrylic and the surrounding tissue is macerated using an alkaline solution. The remaining detailed replication of the vascular network allows for a submicrometer resolution of the cast surface when analysed in the scanning electron microscope, revealing the nature of the blood vessel under investigation through the characteristic endothelial cell imprints of arteries and veins (Miodonski et al., 1976; Murakami, 1971). Vascular corrosion casts can be imaged with high spatial resolution micro tomography (μCT), from which information, including vessel sizes, can be calculated. This method has previously been applied to murine tumour xenografts to investigate tumour vascular structure and the response of tumour vasculature to novel therapeutics (Abbas et al., 1990; El Emir et al., 2007; Ferretti et al., 2005; Folarin et al., 2010; Malcontenti-Wilson et al., 2001; O'Connor et al., 2009; Salmon et al., 2006). Vascular corrosion casts therefore offer an attractive method of validation of both R_v_ and fBV.

The clinical development of novel tumour vascular targeted therapeutics requires the development of more specific non-invasive biomarkers of disrupted tumour vasculature, as afforded by R_v_ and fBV. Such imaging biomarkers require pre-clinical evaluation before being deployed in clinical trials (Waterton and Pylkkanen, 2012). In this study, susceptibility contrast MRI derived values of R_v_ and fBV were compared with corresponding measurements from μCT images of vascular corrosion casts obtained from the same SW1222 colorectal cancer xenografts. In addition, to assess the sensitivity of R_v_ to changes in the distribution of tumour blood vessel calibre, SW1222 tumours were treated with the vascular disrupting agent ZD6126 in order to provide a perturbation of tumour vascular structure. ZD6126 has been shown to cause massive central necrosis in a range of solid murine xenograft models 24 h after treatment, caused by acute and catastrophic vascular collapse (Blakey et al., 2002; Robinson et al., 2003a). Perturbations in the distribution of blood vessel calibre post treatment with ZD6126 can be measured from μCT images of vascular corrosion casts, thereby providing a powerful histological validation of the sensitivity of R_v_ to a vascular intervention.

## Materials and methods

### Cell culture

Human SW1222 colorectal tumour cells were cultured in Dulbecco's Modified Eagles Medium (DMEM; Invitrogen, Paisley, UK) supplemented with 10% (v/v) foetal bovine serum, 5 mM l-glutamine, 100 IU/ml penicillin and 100 μg/ml streptomycin (all Invitrogen). Cells were maintained at 37 °C in a humidified 5% CO_2_ atmosphere.

### Tumour propagation

All experiments were performed in compliance with licences issued under the UK Animals (Scientific Procedures) Act 1986 following local ethical review, and with the United Kingdom National Cancer Research Institute guidelines for the welfare of animals in cancer research (Workman et al., 2010). Female NCr nude mice, under isoflurane anaesthesia, were injected subcutaneously on the right flank with 5 × 10^6^ SW1222 cells. The SW1222 tumours were imaged approximately two weeks after inoculation, when they had reached an average diameter of 1 cm.

### Formulation of ZD6126

ZD6126 (prodrug of N-acetylcolchinol, Angiogene, UK) was formulated in 20% of 5% sodium carbonate and 80% phosphate-buffered saline, yielding a clear solution at pH 7.

### Vessel size index (VSI) MRI protocol

Prior to MR imaging, anaesthesia was induced by an intraperitoneal injection of 10 ml/kg of fentanyl citrate (0.315 mg/ml) plus fluanisone (10 mg/ml) (Hypnorm, Janssen Pharmaceutical, High Wycombe, UK), midazolam (5 mg/ml) (Hypnovel, Roche, Burgess Hill, UK), and water (1:1:2). A lateral tail vein was cannulated with a 27G butterfly catheter (Venisystems; Hospira, Royal Leamington Spa, UK) for remote administration of USPIO particles (ferumoxtran-10, Sinerem®, Guerbet, Roissy, France). During imaging all mice were restrained using dental paste in order to limit motion artefacts (Landuyt et al., 2002). A warm air blower was used to maintain the animal's core temperature at 37 °C.

Mice were imaged 24 h after an i.p. injection of 30 (n = 6) or 200 mg/kg (n = 3) ZD6126, or vehicle alone (n = 6). All images were acquired on a 7 T horizontal bore microimaging system (Bruker, Ettlingen, Germany) using a 3 cm birdcage coil. Field homogeneity was optimised over the entire tumour volume using FASTMAP. Following tumour localisation using a set of T_2_-weighted TurboRARE images, diffusion weighted spin-echo (DWSE, 6 b-values ranging from 6 to 504 s/mm^2^ in the read direction, gradient strength = 0 to 0.1 T/m, gradient duration = 5 ms, gradient spacing = 25 ms, T_R_ = 1000 ms, T_E_ = 36 ms), multiple gradient echo (MGE, T_R_ = 300 ms, 8 echo times ranging from T_E_ = 6.1 to 28.2 ms, flip angle = 45°, 16 averages) and two separate sets of spin echo (SE, T_R_ = 3000 ms, 2 echo times of either 8 or 80 ms, flip angle = 90°, 2 averages) images were acquired for determination of the apparent diffusion coefficient (ADC), R_2_* and R_2_ respectively (Walker-Samuel et al., 2012). All images were acquired from three contiguous 1 mm slices through the tumour centre, with identical geometry (for all image acquisitions field of view = 3 × 3cm, matrix size = 64 × 64). A second set of identical MGE and SE images were acquired 2 min after the i.v. injection of 200 μmol/kg ferumoxtran-10, to allow for a steady state distribution of contrast agent throughout the vascular network.

### Calculation of R_v_ (μm) and fractional blood volume (fBV, %)

Regions of interest (ROIs) corresponding to tumour regions, but excluding the skin, were drawn on DWSE, MGE and SE images. Estimates of ADC, R_2_* and R_2_ were evaluated using a Bayesian hierarchical model which calculates the uncertainty associated with each estimate, and allows non significantly enhancing voxels, post USPIO particle injection, to be excluded (Walker-Samuel et al., 2010). MRI relaxation rates R_2_ and R_2_* were calculated from SE and MGE images respectively, as were changes in these MRI relaxation rates, ΔR_2_* and ΔR_2_, post injection of USPIO particles (Walker-Samuel et al., 2012). Tumour fBV and R_v_ were then calculated from ΔR_2_, ΔR_2_*, and ADC, using the following relationships, originally defined in the methods of Yablonskiy and Tropres (Tropres et al., 2001; Yablonskiy and Haacke, 1994).

$$\text{fBV}=\frac{3}{4\text{π}}\frac{{\text{ΔR}}_{2}*}{{\text{γΔχB}}_{0}}$$

$${\text{R}}_{\text{v}}={\left(\frac{\text{ADC}}{{\text{γΔχB}}_{0}}\right)}^{1/2}{\left(\frac{{\text{ΔR}}_{2}*}{{\text{ΔR}}_{2}}\right)}^{3/2}$$

### Microvascular corrosion casting protocol

Immediately after the MRI acquisition, each animal was killed by asphyxiation. Following a midline laparotomy, the ascending carotid and the femoral arteries were ligated using household thread. This step was taken to isolate, and ensure good injection pressure, to the skin. The chest was then opened along the sternum to expose the heart, the ascending aorta isolated, and a ligature passed underneath it. A small puncture was made in the bottom of the heart using micro scissors, and a blunted 25G butterfly cannula was inserted through the heart and into the ascending aorta, where it was secured using the previously positioned ligature that was passed underneath the aorta. Physiological saline (10 ml) was injected through the cannula at 60 ml/h using a syringe driver (Harvard Apparatus, Tonbridge, UK), and a small incision was made in the right ventricle to form an open system, ensuring that blood was flushed from the entire vascular network. Once the saline injection was complete, after approximately 10 min, a monomer resin (Mercox 2, Ladd, Burlington, USA) was mixed with a catalyst to begin the polymerising process (0.3 g catalyst per 10 ml resin), and injected via the same cannula at 60 ml/h until the vascular bed was completely perfused with resin. Mercox is a low viscosity resin which begins to polymerise to form a solid acrylic upon contact with the catalyst. Using 0.3 g of catalyst per 10 ml resin gives a pot life of 10 min, which was sufficient to allow infusion of approximately 9 ml of resin before complete polymerisation, and a sufficient volume to completely perfuse the abdominal viscera and the subcutaneous tumours in mice. Following perfusion, the resin was allowed to completely polymerise, which took approximately 2 h, after which the entire mouse was placed in 7.5% potassium hydroxide solution for 48 h to macerate. Once maceration was complete, the tumours were dissected, cleaned in distilled water, frozen, and finally freeze dried. The freeze drying step prevented the forces that would be exerted on the cast during air drying from damaging delicate microvessel structures. Once the casts were fully prepared, they were mounted on a scanning podium, and μCT images were acquired using a Nikon X-tek XTH225 CT system (Nikon Metrology, X-tek, Tring, UK).

### Calculation of vessel diameter from μCT data

A histogram of vessel diameters was derived from each μCT reconstructed data set using Avizo 6.3 image processing software (VSG Inc., Burlington, MA). The cast was segmented from background using the ‘magic wand’ region growing tool. The AutoSkeleton module was then applied to derive a 3-D skeletonised image from which the distribution, and mean value, of the vessel diameters was calculated using the RadiusHist module.

Prior to the analysis of all casts, the effect of μCT spatial resolution on the calculation of vessel diameters was first investigated by scanning one vascular cast using two tomography systems to give a range of spatial resolutions. The higher resolutions were provided by an Xradia microXCT-400 system (Xradia Inc, Pleasanton, CA), which features a lens-based high resolution detector. Scans were carried out using the 10 × and 4 × objectives, giving projection image pixel sizes of 1.2 and 2.4 μm respectively. In both cases, a source voltage of 40 kV was used, with 1801 projections being taken over 184°. In-line phase contrast was used together with phase retrieval using the TIE-HOM algorithm to improve SNR of the projection images (Bradley et al., 2010; Keyriläinen et al., 2010). A lower resolution absorption contrast scan was taken using the Nikon X-tek XTH225 system, with a source voltage of 47 kV and 2884 projection images being taken over 360°. 3-D volumetric data was derived from the raw projection images via the FDK reconstruction algorithm (Kak and Slaney, 1988). The range of resolutions was extended by down-sampling the 3-D volumetric data, to give voxel sizes of 1.2, 2.4 and 4.7 μm for the Xradia data, and 10.2 and 17.9 μm for the X-tek data. The reconstructed data were then cropped to give the same cast volume in all cases, but at a range of resolutions. Gold beads were placed on the cast to enable the same region to be identified in all cases. For the highest resolution data, the large number of voxels (2048 × 2048 × 2048) made direct application of the skeletonisation method computationally prohibitive. Therefore the data was split into 400 slice thick sections, and the skeletonisation procedure was applied on each separately. The final mean vessel diameter was then calculated as an average over all the sections. Subsequently, all casts were scanned on the Nikon X-tek system, using a 16.4 μm voxel size, corresponding to a minimum detectable radius comparable to that of the MRI measurements at ~ 8 μm.

The VSI MRI approach provides estimates of median tumour vessel diameter using data from three contiguous image slices approximately through the centre of a tumour, whereas the μCT method analyses (in terms of average or median values etc.) vessel diameters from the entire tumour volume. To investigate the effect of this difference in sampled tumour volume on the calculated mean vessel diameter, the μCT vessel diameter measurement method was also applied to three contiguous 1 mm slices through the centre of the 3D reconstruction of two vehicle vascular casts.

### Statistics

All data are reported as mean ± 1 s.e.m., calculated from the standard deviation of each treatment group, unless otherwise stated. Significance testing employed the non-parametric Mann–Whitney *U* test, with a 5% level of significance.

## Results

**Fig. 1:**
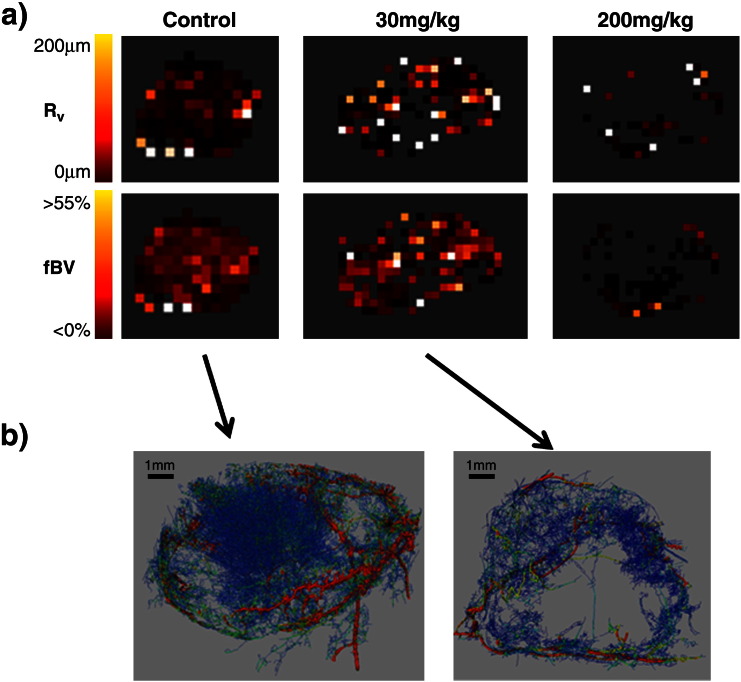
a) Representative parametric R_v_ (μm) and fBV (%) MRI maps acquired from SW1222 colorectal xenografts 24 h after treatment with either vehicle, 30 or 200 mg/kg of the vascular disrupting agent ZD6126. b) μCT images of the corresponding vascular corrosion casts obtained from the same tumours from mice treated with vehicle or 30 mg/kg ZD6126. The images show the skeletonization of the CT data where the colour of the vessels is varied linearly with diameter, from blue ≤ 16.8 µm, to red ≥ 270 µm. The vessel thicknesses/diameters are rendered with reduced scale/size to aid visualization. No μCT data is presented from tumours treated with 200 mg/kg ZD6126, as treatment decreased the fBV so dramatically as to inhibit adequate resin perfusion.

Representative MRI derived R_v_ and fBV maps, and μCT images of microvascular corrosion casts from the same SW1222 xenografts, revealing the relationship of the spatial distribution of blood volume and vessel size, and their response to ZD6126, are shown in Fig. 1. The number of significantly enhancing voxels in the MRI image maps was seen to decrease between vehicle and treated groups, and this effect was more exaggerated in tumours from mice treated with 200 mg/kg than 30 mg/kg ZD6126. Quantitation of the MRI data revealed that, compared to the vehicle group, fBV was lower in tumours treated with either 30 or 200 mg/kg ZD6126, and was highly significant (p < 0.01) in the cohort treated with 200 mg/kg (Table 1). The ZD6126-induced decrease in fBV corresponded with areas of low, or zero vascular density on the μCT images of vascular corrosion casts obtained from the same tumours.

**Fig. 2:**
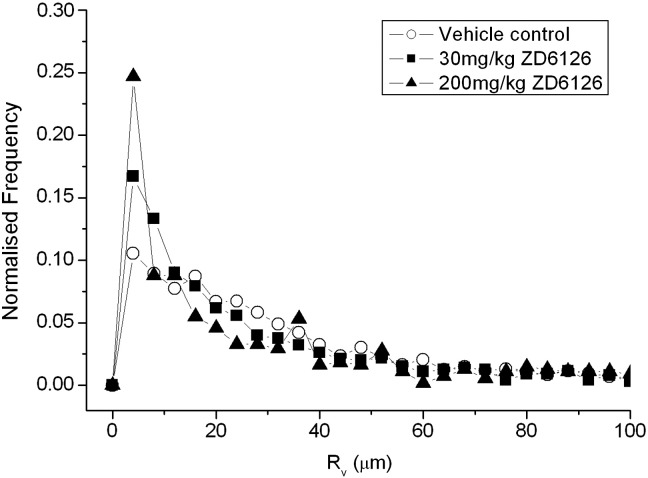
Normalised frequency histogram of R_v_ (μm) values, determined from SW1222 tumours 24 h after treatment with either vehicle (n = 6), 30 mg/kg (n = 6), or 200 mg/kg (n = 3) ZD6126.

No significant difference in R_v_ was found across the treatment groups (Table 1). Plotting the distribution of R_v_ values for each treatment group in a normalised frequency histogram illustrates that only subtle changes in the distribution of R_v_ values were measured with increasing dose of ZD6126 (Fig. 2). The distribution of R_v_ values did not differ between the vehicle, 30, or 200 mg/kg treated tumours, although a slight decrease in the frequency of R_v_ values between 12 and 40 μm is apparent in the cohort treated with 200 mg/kg, compared to the 30 mg/kg and vehicle groups. Table 1 shows the 25th and 75th percentiles of R_v_, calculated from each voxel from every tumour included in the study. The 25th percentile of R_v_ was significantly lower (p < 0.05) in tumours from mice treated with ZD6126. There was no difference in the 75th percentiles between any of the groups.

**Fig. 3:**
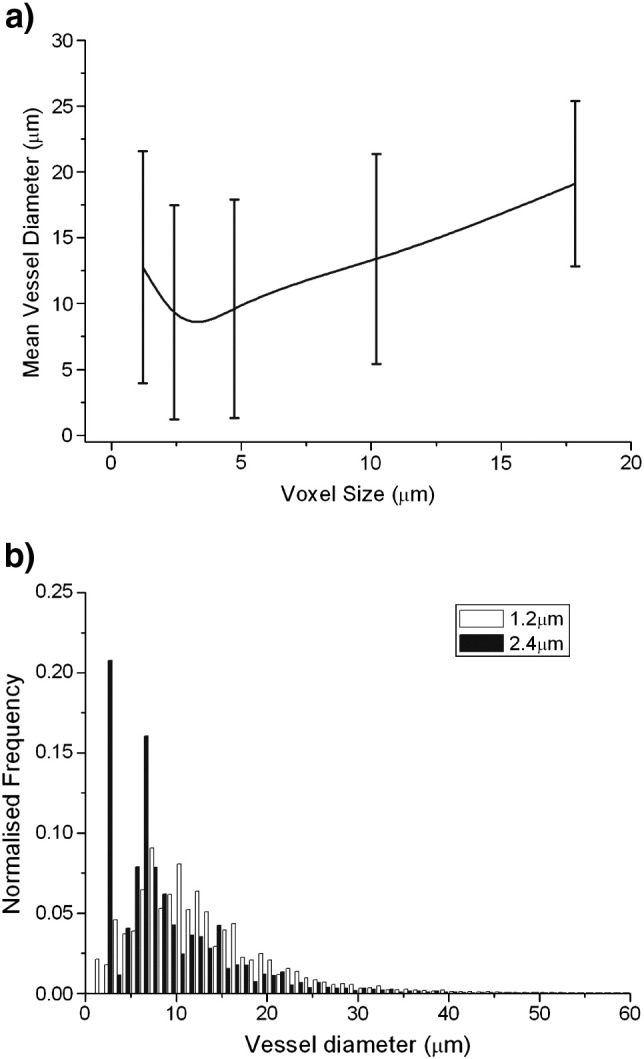
(a) Variation of mean vessel diameter with voxel size of the tomography data. The error bars show the standard deviations calculated from the vessel diameter distributions, and (b) comparison of the distribution of vessel diameters for a voxel size of 1.2 and 2.4 μm.

The variation of calculated mean vessel diameter determined from the μCT data, with reconstructed voxel size, is shown in Fig. 3a, highlighting that as the voxel size was increased, the mean vessel diameter increased. As shown in Fig. 3b, at the highest resolution, the histogram of vessel sizes has only one peak, indicating that there is sufficient resolution to accurately determine the median radius to be 6.3 μm. With decreasing the resolution, a second maximum appears in the histogram at the lowest voxel size. At a voxel size of 16.8 μm, which provides a limit on the smallest detectable vessel similar to that of the MRI technique, the majority of the detected vessels have a diameter at this limit.

Median values of vessel diameter calculated using just three contiguous 1 mm slices through the centre of the 3D reconstructed μCT images of SW1222 vascular casts are shown in Table 2. The corresponding mean vessel diameters, calculated from the entire 3D reconstructed datasets, are shown for comparison. There was no significant difference between median vessel diameters calculated from just the three 1 mm slices, or from the full data.

The vascular casting procedure failed in all of the mice treated with 200 mg/kg ZD6126, a consequence of the extreme reduction in fBV in the SW1222 tumours 24 h after treatment, precluding adequate resin delivery. Corresponding vessel diameter measurements made from μCT images of vascular corrosion casts of the same SW1222 tumours that underwent the VSI imaging protocol are shown in Table 3. There was no significant difference in mean vessel diameter between the vehicle and 30 mg/kg ZD6126 tumour cohorts, in agreement with the R_v_ data. Comparison of the mean μCT and median MRI derived vessel diameters revealed a strong association between the two measurement methods. VSI estimated that median R_v_ diameters were 24 ± 2 and 23 ± 3 μm, and the corresponding μCT measurements from corrosion casts were 25 ± 2 and 28 ± 4 μm, for the vehicle and treated tumours, respectively.

## Discussion

Traditional response evaluation criteria in solid tumours (RECIST) use a reduction in tumour volume as a measure of response to therapy. However, vascular targeted therapies are not predicted to be directly cytotoxic and cause significant tumour regressions, and hence changes in tumour volume are an inappropriate indicator of treatment efficacy. For this reason, non-invasive functional imaging biomarkers are continually being identified and validated for the evaluation of novel therapeutics (Waterton and Pylkkanen, 2012).

Susceptibility contrast MRI with USPIO particles provides quantitative biomarkers of weighted mean vessel diameter (R_v_, μm), and fractional blood volume (fBV, %) (Tropres et al., 2001; Yablonskiy and Haacke, 1994). Quantitation of fBV has been used to assess tumour response to anti-vascular treatment, and the imaging data shown to correlate with appropriate histological measures, such as fluorescence microscopy of the uptake of the perfusion marker Hoechst 33342 (Robinson et al., 2007). Validation of R_v_ has been typically pursued using immunohistochemical detection of pan-endothelial markers such as CD31 or collagen type IV, morphometric analysis of Hoechst 33342 uptake in individual vessels, and two photon laser scanning microscopy methods, to measure vessel diameter from tissue samples (Douma et al., 2010; Howe et al., 2008; Lemasson et al., in press; Remmele et al., 2011; Robinson et al., 2008; Tropres et al., 2004; Valable et al., 2008; Walker-Samuel et al., 2012). A common inference from these studies was that susceptibility MRI tended to overestimate median vessel diameter, but that R_v_ shows significant correlations with histological vessel size measurements for the measurement of differences in vessel diameter distributions. The variance may be a consequence of structural deformations that occur with histological tissue preparation.

In this study, R_v_ was quantified from SW1222 colorectal xenografts, and compared with vessel diameters measured from μCT images of microvascular corrosion casts derived from the same tumours. The R_v_ for control SW1222 tumours was ~ 24 μm, which is similar to that reported in a wide range of subcutaneously propagated xenografts (Douma et al., 2010), and smaller than that determined in orthotopically propagated xenografts (Wade and Kozlowski, 2007; Walker-Samuel et al., 2012). Collectively, these data highlight the importance of site of tumour propagation on the subsequent developing vascular morphology. Importantly, an excellent association between R_v_ and the tumour vessel diameter derived from μCT of the vascular corrosion casts was found, providing strong qualification of the MR imaging biomarker (Waterton and Pylkkanen, 2012).

The vascular disrupting agent ZD6126 was used to perturb the tumour vasculature. Treatment with ZD6126 elicited a dose-dependent reduction in fBV of SW1222 tumours compared to vehicle control, and which was primarily associated with the tumour core. Similar decreases in fBV following treatment with vascular disrupting agents have been reported in other tumour models (Bradley et al., 2007; Robinson et al., 2007; Vogel-Claussen et al., 2007; Walker-Samuel et al., 2012). Interestingly, there was no associated difference in R_v_ between vehicle and ZD6126 treated tumours, and which was confirmed by μCT imaging of the corrosion casts. Together this suggests that treatment with ZD6126 significantly reduced functional tumour vasculature, but had no effect on the distribution of vessel calibre. It could thus be hypothesised that ZD6126 can indiscriminately target both immature small blood vessels, and more mature larger vessels.

Histogram analysis of the distribution of R_v_ can provide a deeper interrogation of susceptibility contrast MRI data (Howe et al., 2008; Just, 2011; Walker-Samuel et al., 2012). The histograms in Fig. 3 illustrate that, although there was no difference in the median value of R_v_ in any of the tumour cohorts, values from all groups exhibited a different shaped distribution in the range of 12 to 44 μm. This difference was most pronounced in the ZD6126 treated cohorts, suggesting that treatment influenced the distribution of vessel diameters in this range. Accordingly, the 25th percentiles of R_v_ were significantly lower in tumours treated with ZD6126 compared to vehicle controls. Previous studies have reported similar decreases in the 25th percentile of R_v_ in response to other vascular disrupting agents (Howe et al., 2008; Walker-Samuel et al., 2012). Collectively, these data suggest that the 25th percentile may be a more sensitive measure of changes in vessel calibre than the median.

This study also describes optimisation of approaches for μCT imaging of microvascular corrosion casts. An important factor was found to be the μCT resolution. As the μCT imaging voxel size used for image acquisition was increased, there was an associated increase in vessel diameter (Fig. 3). Data acquired using a 1.2 μm voxel showed a single maximum, whereas a second peak appeared when the voxel size was doubled to 2.4 μm. This arises from partial volume effects, where the grey level of a voxel is increased above the background level by the presence of a vessel contained within it, even though the vessel itself cannot be fully resolved. As the voxel size increases from 1.2 μm and approaches the radii of the smallest capillaries, around 6 μm, there is a decrease in mean vessel size, as the secondary maximum is at a lower radius than the ‘true’ maximum. Once the voxel size becomes greater than the smallest true vessel diameter the calculated mean diameter increases with voxel size. The results therefore suggest that a voxel size of less than 2 μm is required to accurately determine the mean and vessel diameter. However, at this resolution, the reconstructed volume is only ~ 30 mm^3^, and therefore over 20 scans would typically be required from each cast to determine the vessel calibre over the entire cast. Alternatively, the cast could be sampled with a smaller number of scans; however care would have to be taken to ensure that the samples were representative given the heterogeneous spatial distribution of vessels. At lower μCT resolutions, despite overestimation, the median and diameters would still be representative of the vessels that can be detected at that resolution and hence indicative of the relative proportions of small (at or around the spatial resolution limit) and larger vessels within the cast. Thus, the results from different vascular casts can still be compared, provided that the same resolution is used in all cases. Therefore, a voxel size of 16.4 μm, corresponding to a minimum detectable radius comparable to that of the MRI measurements at ~ 8 μm was used, enabling the whole cast to be analysed from one scan (Tropres et al., 2001).

There was no significant difference between median vessel diameters calculated using just three contiguous 1 mm slices through full 3D rendered μCT images of the vascular corrosion casts, and mean vessel sizes measured from the whole tumour μCT images. There appeared thus to be no added value in attempting to spatially match the μCT measurements of vessel diameter from vascular corrosion casts with the slice positions of the MRI images. Additionally, analysing the distribution of vessel sizes in three 1 mm slices through a large 3D μCT dataset is less time consuming than analysing a whole 3D image.

To conclude, despite a significant decrease in fractional blood volume, no decrease in mean MRI-derived vessel calibre (R_v_) was determined in subcutaneously propagated SW1222 colorectal xenografts 24 h after treatment with ZD6126. A significant decrease in the 25th percentile of R_v_ with treatment was determined, suggesting predominant anti-vascular effects on relatively smaller calibre vessels. A strong association was found between R_v_ and mean vessel diameters measured from μCT images of microvascular vascular corrosion casts generated from the same SW1222 tumours immediately following the MRI acquisition, providing a powerful technique for the validation of changes in tumour R_v_ following anti-vascular treatment.

## Figures and Tables

**Table 1:** Summary of the quantitative MRI data. MRI derived fractional blood volume (fBV, %) and weighted vessel diameter (R_v_, μm) measurements determined from SW1222 xenografts 24 h after treatment with either vehicle, 30 or 200 mg/kg ZD6126. Data are median, and mean ± 1 s.e.m. of the median values obtained from each imaged slice for each tumour, ^#^p = 0.09, **p < 0.01, one-tailed Mann–Whitney test. Also shown are the mean 25th and 75th percentiles of R_v_. Data are mean ± 1 s.e.m, *p < 0.05, two-tailed Mann–Whitney test.

	Vehicle (n = 6)		30 mg/kg (n = 6)		200 mg/kg (n = 3)	
	fBV	R_v_	fBV	R_v_	fBV	R_v_
Median	13.5	24.4	12.3	21.2	1.8	20.3
Mean	13.2 ± 1	24.3 ± 2	11 ± 1^#^	23.3 ± 3	2 ± 0.5**	23.5 ± 6
25th percentile		12 ± 2		7 ± 1*		6 ± 2*
75th percentile		58 ± 10		80 ± 8		59 ± 26

**Table 2:** Comparison of median vessel diameters (μm) calculated from just three contiguous 1 mm slices through the centre of the full μCT rendering of SW1222 vascular corrosion casts obtained from two vehicle treated mice, and the mean vessel diameters calculated from the full 3D reconstructed μCT images. Values are median or mean ± standard deviation.

	Slice 1	Slice 2	Slice 3	Median	Mean (whole tumour)
Cast 1	22 ± 16	23.6 ± 20	21.8 ± 14	22.4 ± 1	24 ± 26
Cast 2	21.6 ± 30	18.2 ± 8	17.4 ± 16	19 ± 1	18.4 ± 14

**Table 3:** Mean vessel diameter measured from μCT images of microvascular corrosion casts of SW1222 xenografts 24 h after treatment with either vehicle (n = 4) or 30 mg/kg ZD6126 (n = 5). Data are mean ± 1 s.e.m. of the values obtained from each imaged cast.

	Vehicle (n = 4)	30 mg/kg (n = 5)
Mean vessel diameter (μm)	24.5 ± 2	28.4 ± 4
